# *DPYD* and Fluorouracil-Based Chemotherapy: Mini Review and Case Report

**DOI:** 10.3390/pharmaceutics11050199

**Published:** 2019-05-01

**Authors:** Theodore J. Wigle, Elena V. Tsvetkova, Stephen A. Welch, Richard B. Kim

**Affiliations:** 1 Department of Physiology and Pharmacology, Schulich School of Medicine & Dentistry, Western University, London, ON N6A 3K7, Canada; twigle@uwo.ca; 2 Division of Clinical Pharmacology, Department of Medicine, Schulich School of Medicine & Dentistry, Western University, London, ON N6A 3K7, Canada; 3 Division of Medical Oncology, Department of Oncology, Schulich School of Medicine & Dentistry, Western University, London, ON N6A 3K7, Canada; elena.tsvetkova@lhsc.on.ca (E.V.T.); stephen.welch@lhsc.on.ca (S.A.W.)

**Keywords:** dihydropyrimidine dehydrogenase, *DPYD*, 5-fluorouracil, fluoropyrimidine, therapeutic drug monitoring, orthotopic liver transplant

## Abstract

5-Fluorouracil remains a foundational component of chemotherapy for solid tumour malignancies. While considered a generally safe and effective chemotherapeutic, 5-fluorouracil has demonstrated severe adverse event rates of up to 30%. Understanding the pharmacokinetics of 5-fluorouracil can improve the precision medicine approaches to this therapy. A single enzyme, dihydropyrimidine dehydrogenase (DPD), mediates 80% of 5-fluorouracil elimination, through hepatic metabolism. Importantly, it has been known for over 30-years that adverse events during 5-fluorouracil therapy are linked to high systemic exposure, and to those patients who exhibit DPD deficiency. To date, pre-treatment screening for DPD deficiency in patients with planned 5-fluorouracil-based therapy is not a standard of care. Here we provide a focused review of 5-fluorouracil metabolism, and the efforts to improve predictive dosing through screening for DPD deficiency. We also outline the history of key discoveries relating to DPD deficiency and include relevant information on the potential benefit of therapeutic drug monitoring of 5-fluorouracil. Finally, we present a brief case report that highlights a limitation of pharmacogenetics, where we carried out therapeutic drug monitoring of 5-fluorouracil in an orthotopic liver transplant recipient. This case supports the development of robust multimodality precision medicine services, capable of accommodating complex clinical dilemmas.

## 1. Introduction to Fluoropyrimidines

5-fluorouracil (5-FU) has remained an important antineoplastic agent since the first description of the fluoropyrimidine class in 1957, and approval for testing in humans in 1962 [1,2]. Fluoropyrimidines, including 5-fluorouracil and its oral pre-prodrug capecitabine, serve as core components in the treatment of colorectal, pancreatic, gastric, breast, head and neck cancers [2,3,4]. However, the use of fluoropyrimidines carries an unfortunate risk of severe adverse events (AEs) of up to 30% [5,6]. Common AEs observed with fluoropyrimidine chemotherapies include non-bloody diarrhea, mucosal ulceration, immune suppression, and a painful skin condition known as hand-foot syndrome. Through optimizing the delivery methods, dosing schedules, and concomitant antineoplastic agents, a number of modern combination regimens with a fluoropyrimidine backbone have emerged including FOLFOX, FOLFIRINOX, CAPOX, and FLOT. Nevertheless, clinical trials using fluoropyrimidine-based chemotherapies continue to show severe AE rates up to 23% [7,8,9,10]. Accordingly, delineating the genetic and non-genetic determinants of fluoropyrimidine metabolism and efficacy, is essential to the implementation of precision medicine approaches for fluoropyrimidine-based chemotherapy.

**Figure 1:**
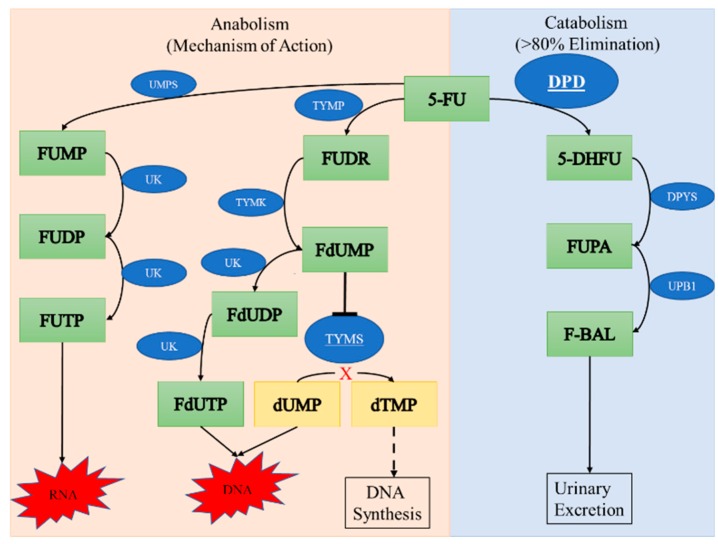
A simplified metabolism of 5-fluorouracil (5-FU). Thymidylate phosphorylase (TYMP) generates fluorouridine (FUDR), which is converted to Fluoro-deoxyuridine monophosphate (FdUMP) by thymidylate kinase (TYMK). FdUMP inhibits thymidylate synthase (TYMS) causing an imbalance of deoxyuridine monophosphate (dUMP) and deoxythymidine monophosphate (dTMP). Incorporation of dUMP into DNA causes damage and leads to cell death. 5-FU is converted to fluorouridine monophosphate (FUMP) by uridine monophosphate synthetase (UMPS) with further phosphorylation by uridine kinase (UK). Incorporation of fluorinated nucleotides (FUTP or FdUMP) into both RNA and DNA respectively leads to cell death. Inactivation of 5-FU occurs through dihydropyrimidine dehydrogenase (DPD) conversion to 5-dihydrofluorouracil (5-DHFU). Dihydropyrmidinase (DPYS) catalyzes the creation of fluoro-beta-ureidopropionate (FUPA) and beta-ureidopropionase (UPB1) activity culminates in urinary elimination of fluoro-beta-alanine (FBAL).

Fluoropyrimidines are an antimetabolite class of chemotherapeutic. As antimetabolites, they target replicating cells. Fluoropyrimidines act primarily through conversion of 5-FU to fluoro-deoxyuridine monophosphate (FdUMP). FdUMP acts as an irreversible inhibitor of the thymidylate synthase enzyme this is stabilized by forming a ternary complex with the reduced folate species methylene-tetrahydrofolate. Thymidylate synthase plays an important role in regulating the nucleotide pool by converting deoxyuridine monophosphate (dUMP) to deoxythymidine monoposhpate (dTMP), providing this pyrimidine building block for DNA synthesis. When thymidylate synthase is inhibited, the buildup of dUMP nucleotides leads to their incorporation into DNA, which overwhelms DNA repair mechanisms and eventually leads to cell-death. Thymidylate synthase inhibition is the major canonical mechanism of action of fluoropyrimidines, in addition they can exert antineoplastic effects through at least two additional pathways. First, active fluoropyrimidine (FdUMP) can also be incorrectly incorporated into DNA in place of dTMP leading to both single strand and double strand breaks. The resultant DNA damage induces cell cycle arrest and death. Second, 5-FU is converted to fluorouridine triphosphate (FUTP) and incorrectly incorporated in RNA. The combination of RNA damage, DNA damage, and inhibition of cell cycle provide the mechanistic basis for the antineoplastic effects of fluoropyrimidines (Figure 1, for review see [11]). The antimetabolite properties of 5-FU support the antineoplastic effects, but a lack of specificity underpins the AEs seen with this therapy. The classic fluoropyrimidine toxicities occur in rapidly regenerating tissues such as the mucosal membranes, skin, and bone marrow. Therefore, the effective but nonspecific nature of fluoropyrimidines is the likely culprit for numerous AEs. Appropriately balancing the therapeutic benefit vs. toxicity of this class of antineoplastic drugs has proved to be a major challenge, requiring a detailed understanding of the pharmacology.

## 2. Metabolism and Clearance of 5-FU

Understanding the metabolism of 5-FU took nearly 35 years to flesh out. Heidelberger and colleagues knew from early stages that 5-FU was rapidly metabolized [1,12], and from human pharmacologic studies we now know that 5-FU has a half-life ranging from 8 to 20 min, varying with route of administration [13]. Heidelberger and colleagues were unable to completely parse out the different effects of the catabolic and anabolic pathways on 5-FU metabolism. The anabolic pathway is directly related to the fluoropyrimidine mechanism of action through generation of FdUMP, and initially it was believed this pathway was also responsible for the elimination of 5-FU. However, the earliest studies were limited by the sensitivity of available analytical assays and the rapid degradation of 5-FU metabolites, thereby producing conflicting results [14]. With the development of a new high-pressure liquid chromatography technique, researchers were then able to accurately measure 5-FU metabolites [15]. These studies confirmed dihydropyrimidine dehydrogenase (DPD; EC 1.3.1.2, encoded by *DPYD*) to be the first and rate-limiting enzyme in the catabolic cascade of 5-FU, but did not establish the clinical significance of these findings [15,16]. The pivotal role of DPD activity in fluoropyrimidine catabolism and the implications of DPD deficiency for fluoropyrimidine-related AEs were identified in the clinical literature shortly thereafter. The first case report presented a patient treated with 5-FU who had oral ulceration, neurotoxicity, and severe myelosuppression leading to hospitalization. This patient, and a first degree relative, were found to have familial pyrimidinemia and pyrimidinuria—characterized by elevated uracil and thymine in both blood and urine [17]. This first case report provided the initial link between an inborn error of metabolism and fluoropyrimidine-related AEs. While Tuchman et al. were not able to directly assess DPD activity in this patient, a key corollary of their findings is the knowledge that the endogenous function of DPD is the metabolism of both uracil and thymine [18]. Within two years, a pharmacokinetic analysis of 5-FU metabolism in cancer patients demonstrated that the primary process of 5-FU elimination occurred through DPD-dependent catabolism. This study found that the catabolic pathway is responsible for the elimination of over 80% of systemic 5-FU, with 95% of the final metabolite being eliminated in the urine (Figure 1) [19]. Following this confirmation of DPD as the key metabolic enzyme responsible for 5-FU elimination, Diasio et al., published a case report of severe fluoropyrimidine induced neurotoxicity in a female patient with familial DPD deficiency. This patient also developed profound neutropenia requiring hospitalization [20]. There were a number of case reports that followed this publication and supported the link between DPD deficiency and fluoropyrimidine toxicities [21,22,23]. Cloning of *DPYD* set the stage for identifying the molecular basis of this hereditary defect [24]. It was identified that the most common familial DPD deficiency was linked to a defect in processing of the DPD precursor mRNA, namely an exon skipping variant resulting in the loss of 165 nucleotides from the fully spliced mRNA [25]. However, the first paper to identify the mechanistic cause of the deficiency failed to identify the point mutation responsible for this effect. The first DNA sequence level identification of this *DPYD* variant was published one year later by two different groups one-month apart, they presented the same findings in two unrelated families. They identified a single nucleotide polymorphism (SNP) within *DPYD* that introduced a new splice site, which resulted in exon 14 skipping. The resultant DPD protein has complete loss of function [26,27]. This variant is now commonly referred to as DPYD*2A (also known as: c.1905+1G>A or rs3918290) and plays a major role in driving research of the pharmacogenetic influences of fluoropyrimidine-related AEs. The DPYD*2A allele is present in approximately 2% of Caucasians of European descent. Heterozygous carriers of this allele exhibit a 50% reduction of DPD activity. While very rare (~1:1000), homozygous DPYD*2A patients demonstrate complete DPD deficiency [28]. Complete DPD deficiency can remain undetected in otherwise healthy individuals. Unfortunately, the consequences of unrecognized DPD deficiency during fluoropyrimidine chemotherapy can be lethal. To this day there continue to be case reports of the lethal consequences of fluoropyrimidine chemotherapy in completely DPD deficient patients [29,30]. This disquieting reality of fluoropyrimidine chemotherapy has led to many efforts to understand and implement pre-treatment screening for DPD deficiency.

## 3. Understanding DPD Activity

Given the association between DPD deficiency and severe fluoropyrimidine-related AEs, there is a requirement to understand the baseline variation in DPD activity. It was first identified that DPD activity follows a circadian rhythm: DPD activity peaks near midnight, with trough DPD activity in the early afternoon [31]. This curious discovery was linked to variation in the systemic 5-FU exposure during prolonged continuous infusions, with a change in systemic 5-FU levels from peak to trough of 2.3-fold during a prolonged 5-day course [32]. However, there is little agreement on the value of predicting this chronological rhythm, or the rhythm’s physiologic significance [33,34,35]. In general, it is now understood that hepatic DPD activity is responsible for the majority of 5-FU clearance [13], and on a population level follows a normal distribution [36]. Lu et al., quantified DPD activity from frozen liver sections using a radiolabeled biochemical assay. The authors showed a strong correlation between DPD protein level expression and enzyme activity [36]. Alternative attempts to quantify DPD activity sought to correlate mRNA expression with DPD activity. Initial studies showed a strong correlation between DPD mRNA and DPD activity in *DPYD* wild-type individuals [37,38]. However, eventually, this line of study was abandoned as it was realized that increased expression of mRNA would not compensate for a functionally inactive enzyme. Therefore, DPD mRNA levels would not be reflective of global DPD activity or provide sufficient understanding of 5-FU elimination. Given DPD is widely expressed, researchers have sought to understand population variation in DPD activity through the study of peripheral blood mononuclear cells (PBMCs). There was a significant but limited correlation between PBMC DPD activity and hepatocyte DPD activity R^2^ < 0.6 [39,40], which makes interpreting the relevance of PBMC DPD activity studies more challenging. In addition, the correlation between PBMC DPD activity and systemic 5-FU clearance demonstrated even weaker associations than between PBMC DPD activity and hepatic DPD activity [39,41]. Finally, PBMC DPD activity demonstrated greater variation than was found in studies of hepatic DPD activity, where PBMC DPD activity demonstrated variation of activity between 8- to 21-fold depending on the study [41,42,43,44]. Therefore, the utility of PBMC DPD activity in characterizing the population variation of endogenous DPD activity remains difficult to interpret. In addition, DPD activity is known to differ between healthy and malignant tissues of the same organ [45]. The discrepancies between DPD activity in malignant neoplasms, inflamed mucosa and healthy tissue has led to some debate regarding which tissue type is of greatest importance for DPD activity during fluoropyrimidine chemotherapy. One branch of research chooses to focus on DPD activity in the malignant cells as a predictor of fluoropyrimidine efficacy [37,38,46]. The complimentary studies aim to interrogate global DPD activity, as a more relevant variable in the systemic clearance of 5-FU and therefore fluoropyrimidine-related AEs [47]. 

## 4. Evolution of DPD Activity Testing

The primary goal for pre-treatment DPD activity assessment is to accurately predict patients with deficient clearance of fluoropyrimidines who are at an increased risk for severe AEs. Given the predominantly hepatic catabolism of systemic fluoropyrimidines, pre-treatment testing needs to approximate the baseline status of hepatic metabolism. While liver biopsy for DPD activity determination has been performed experimentally [36] it is not a reasonable approach for scaling as a pre-treatment screening tool. Therefore, peripheral measurement of DPD activity has been pursued as a surrogate for hepatic metabolism. Most early studies focused on biochemical assays of DPD activity in PBMCs. The technique provided a minimally invasive method of directly assessing basal DPD activity, with high sensitivity [48]. However, this method has not garnered wide spread support due to several limitations. First as previously noted, there was a poor correlation between PBMC DPD activity, hepatic DPD activity and systemic 5-FU clearance casting doubt on the clinical relevance of this method [49]. As well incorporating this technique within clinical care is cumbersome for testing laboratories and requires significant infrastructure costs [48]. Therefore, alternative methods for assessing DPD activity have been developed. Endogenous metabolites of DPD activity could provide a physiologically relevant biomarker of DPD activity. With this premise in mind a number of studies have attempted to characterize systemic uracil and dihydrouracil concentrations as endogenous markers of DPD activity [33,35,50,51,52,53,54,55,56,57,58,59,60,61]. DPD converts uracil to dihydrouracil, thus the ratio of product: metabolite could serve as a marker of basal enzyme activity [18,62]. The techniques employed in testing this approach have evolved from labor intensive techniques such as metabolite challenges [55,61], to the pragmatic direct measurement of baseline plasma uracil concentration [59]. The assessment of pre-treatment uracil and dihydrouracil in plasma samples has produced promising results. These tests do not require as extensive an infrastructure and demonstrate predictive value for fluoropyrimidine-related AEs [53,60,63,64]. However, pre-treatment uracil concentration or the dihydrouracil: uracil ratio, has not yet been prospectively validated for predictive use. A recent prospective cohort study employing the dihydrouracil: uracil ratio as a component of a multiparametric pre-treatment testing approach, was unfortunately cancelled early due to safety concerns [65]. Another prospective validation will be completed as a secondary analysis of a recently completed trial of pretreatment *DPYD* genotyping in the Netherlands (clinicaltrials.gov, NCT02324452) [10]. We await the results of this trial and confirmatory results before suggesting the clinical validity of this test in the pre-treatment setting. All of the above assessments of DPD activity are still complicated by the known circadian rhythm of DPD activity. First, researchers need to establish the time of day that is an appropriate reference of global DPD activity. While many attempts have been made to assess the value of using chronicity in fluoropyrimidines, the field remains in a state of flux [49]. In summary, the predictors of fluoropyrimidine-related AEs described thus far have relied upon the direct phenotypic determination of DPD activity; however, a more comprehensive genetic approach may be of a significant clinical benefit. 

There has been a parallel and often intertwined field of study testing the genetic variation within *DPYD* for clinical relevance. After the initial discovery of DPYD*2A there have been numerous studies identifying additional *DPYD* variants and testing for their association with severe AEs in fluoropyrimidine therapy. Since this field was in its infancy during the 1990s, it has been understood that a single genetic variant could not account for the observed frequency of DPD deficiency in the population [21]. Currently there are over 200 *DPYD* variants that have been identified [66]. Leaders in this field have attempted to characterize the effects of many of these variants on DPD activity in vitro, to identify those that are clinically relevant [28,67,68]. This research has supported large scale association studies providing the basis for our current understanding of the field [69,70,71,72,73,74,75,76]. Through a series of systematic meta-analyses researchers have begun to validate the currently actionable *DPYD* variants [77,78,79]. As of 2011, the Royal Dutch Association for the Advancement of Pharmacy’s ‘Pharmacogenetics Working Group’ published guidelines cautiously recommending fluoropyrimidine dose reductions for 14 *DPYD* variants [80]. This guideline has been improved upon and there is now an expert consensus guideline by the Clinical Pharmacogenomics Implementation Consortium (CPIC) that has limited the number of variants to only those with strong supporting evidence [81]. Therefore, the CPIC guideline for *DPYD* and fluoropyrimidines only states four *DPYD* variants as clinically actionable [28]. 

The four variants currently considered clinically actionable include DPYD*2A, DPYD*13, *DPYDc*.2846A>T, and *DPYD* haplotype-B3. We have previously discussed the discovery and characterization of first variant DPYD*2A in DPD deficient patients [26,27]. Through in vitro assessment of DPD enzymatic activity, it has been shown that DPYD*2A leads to complete loss of DPD enzymatic activity [67]. In addition, numerous clinical studies have supported the association between the DPYD*2A variant and fluoropyrimidine-related AEs [72,74,75,82,83,84]. Given the observed complete loss of function and the known association with toxicity the recommendation of a 50% dose reduction was developed [28,81]. Prospective DPYD*2A genotyping with dose reduction was also shown to reduce fluoropyrimidine-related AEs, while attaining cost-effectiveness [9]. The second clinically actionable variant is DPYD*13 (also known as *DPYDc*.1679T>G, or rs55886062, or DPD p.I560S). DPYD*13 was initially discovered through exploratory sequencing of a subset of *DPYD* exons in a single patient with known DPD deficiency [85]. The DPYD*13 variant causes a serine for isoleucine substitution in a highly conserved region of the DPD protein. The interpretation of this change suggests the substitution of a hydrophilic base into an otherwise well-conserved hydrophobic region could lead to destabilizing the protein [82]. In vitro assessment of the DPYD*13 variant demonstrated near complete ablation of DPD enzymatic activity [67]. In Caucasian populations this variant is very rare [28]. This has made the clinical associations for this variant more challenging, however in samples with sufficient power and a meta-analysis it is possible to confirm the DPYD*13 variant is associated with an increased risk for toxicity [71,78,79]. The third actionable *DPYD* variant is *DPYDc*.2846A>T (also known as rs67376798, or DPD p.D949V) was also first identified through exploratory sequencing of *DPYD* exons, in patients that had experienced severe fluoropyrimidine-related AEs [82]. The substitution of valine for aspartic acid at position 560 is proposed to impact the interaction between DPD and its co-factors [82]. The in vitro functional assessment of *DPYDc*.2846A>T shows a 40–60% reduction in enzyme activity [67,68,86]. The partial loss of function is an important distinction between this variant and both DPYD*2A and DPYD*13. The partial reduction in function could alter the potential pharmacogenetic influence of *DPYDc*.2846A>T on fluoropyrimidine-related toxicities. However, there is substantial evidence linking *DPYDc*.2846A>T with increased fluoropyrimidine-related toxicities and the presence of *DPYDc*.2846A>T [69,72,74,75,78,79]. In the original guidelines carriers this variant was recommended to receive a 50% dose reduction of fluoropyrimidines [81]. However, when the guidelines were updated the dose recommendation was changed to state between 25–50%, to account for the functional data highlighting there is not a complete loss of function with this variant [29]. This may change again following recent data suggesting that 25% dose reduction does not sufficient reduce the risk for fluoropyrimidine-related AEs [10]. The fourth *DPYD* variant that is included in the updated pharmacogenetic guidelines is *DPYD* haplotype-B3 (also known as *DPYDc*.1129-5923C>G, or *DPYDc*.1236G>A, or rs75017182 or rs56276561). This haplotype was initially identified by Amstutz et al., in patients with fluoropyrimidine-related toxicities [87]. The characterization of this variant revealed that the variant reduces the mRNA splicing efficiency by 30%. This reduction in functional mRNA production was linked to a 35% reduction in DPD enzymatic activity [88,89]. As with *DPYDc*.2846A>T, *DPYD* haplotype-B3 is an incomplete loss of function with the same inherent implications for the pharmacogenetic relevance of this variant. However, combining the in vitro data with multiple clinical association studies the consensus opinion is that there is sufficient evidence to support *DPYD* Haplotype-B3 as an actionable variant [29,79]. Together these four variants form the base of the current pharmacogenetic guidelines for Caucasian populations. Building upon the consensus CPIC guidelines are strong prospective trials of *DPYD* genotype-guided dosing in fluoropyrimidine therapy, both demonstrating a reduction of severe fluoropyrimidine-related AEs while maintaining cost effectiveness [9,10,90]. This represents a major advancement in the field of pharmacogenetics and supports the wide spread implementation of *DPYD* genotyping pre-treatment. 

Despite the recent advances in the pharmacogenetics of fluoropyrimidine-related AEs, the use of *DPYD* genotyping also has its limitations. When including the four actionable SNPs the sensitivity for severe AEs remains low and accounted for at most 30% of AEs [81], meaning that the causes of many AEs are unaccounted for by genotype testing alone. Furthermore, pharmacogenetic testing has not been widely accepted or recommended as a routine test in the pre-treatment period. While governing agencies concede the danger of fluoropyrimidines in DPD deficient patients they fail to recommend or require pre-treatment DPD testing as a routine test [2,3,91]. In part, the lack of uptake can be traced to concerns over which populations can benefit from the available knowledge, which fluoropyrimidine-containing regimens should be screened, a need for confirmatory cost-analysis, and the current lack of prospective survival outcomes data [92]. Retrospective studies have attempted to address these limitations, showing positive support for both the broad implementation of *DPYD* genotyping in various fluoropyrimidine regimens and non-inferiority in survival outcomes [93,94]. However, further prospective confirmatory studies are required to change the opinion of regulatory authorities. As well there have been important lessons learned from centers that have implemented pre-treatment testing. At our medical center, implementation of *DPYD* genotype testing started with a handful of patients referred to our Personalized Medicine Clinic after severe fluoropyrimidine-related AEs. It was clear from this early implementation that patients who exhibited severe toxicity were far more likely to be carriers of loss of function genetic variants in *DPYD* ~17% compared to the local population frequency ~4%. In the past 5 years, guidelines on the clinical implementation of *DPYD* genotype testing, along with recommended dose reduction have allowed for *DPYD* genotype-guided dosing to be more broadly and confidently provided to requesting physicians. At our center, pre-treatment *DPYD* genotype testing is incorporated into routine care through a prospective cohort study of pharmacogenetic technologies. A multidisciplinary team, including physicians, pharmacists, and nurses work together to provide *DPYD* genotype testing results within 24–48 h after the patient’s initial assessment. Indeed, the ability to provide timely patient centered precision medicine, without delay in treatment timelines has been viewed as highly desirable and beneficial for patient care. Moreover, we now see a clear benefit of pre-treatment *DPYD* testing for preventing severe toxicity as well as cost-effectiveness. In a recent commentary, authors with 8-years experience of providing pre-treatment testing advocate for a multimodality approach to improve the sensitivity and eliminate some of the ambiguity of the *DPYD* genotype testing alone [95]. The concept of a multimodality genotype-phenotype approach has also been incorporated in recent guidelines by the Group of Clinical Pharmacology in Oncology (GPCO)-UNICANCER and the French Network of Pharmacogenetics [96]. Overall, there is strong evidence for the use of *DPYD* genotyping in the pre-treatment setting to reduce the risk of fluoropyrimidine-related AEs. However, this testing alone will not identify all patients with DPD deficiency and additional modalities should still be considered to further improve patient safety outcomes.

## 5. Therapeutic Drug Monitoring for 5-Fluorouracil

We have discussed a few of the known benefits and challenges, implicit in the use of DPD deficiency prediction for fluoropyrimidine-based therapy. Given the limited sensitivity of the available pre-treatment techniques, the use of therapeutic drug monitoring (TDM) may play an important role in promoting the safe and efficacious use of fluoropyrimidines. This work is founded on the early clinical pharmacokinetic literature that demonstrated high systemic 5-FU level was correlated with both disease response and toxicity [97,98]. Therefore, if the systemic drug level could be assessed during active treatment and actively feedback to the treating physicians, dose titration may alter the clinical outcomes for these patients. This work spurred efforts at the first 5-FU TDM trial by Santini et al. who used a retrospective control group and were able to show improvements in both disease response and fluoropyrimidine-related AEs [99]. The work by Santini et al. on head and neck cancers was complimented by comparable trials in colorectal cancers [100,101]. Further advancement led to randomized controlled trials in each disease site. Both trials confirmed the value of 5-FU TDM for both efficacy and AE reduction [102,103]. These trials established the first dose titration algorithms to maximize the therapeutic index of 5-FU. There have been many additional smaller studies of TDM in 5-FU summarized by Lee et al. [104]. The positive results from these studies drove the development of commercial products for 5-FU pharmacokinetic guided dose titrations available in the USA and France (My5-FU^®^, Saladax Biomedical Inc.; ODPM Protocol^TM^, Onco Drug Personalized Medicine). Having analyzed post-marketing data of a commercial assay, Kaldate et al. provided an updated dosing algorithm with a more accessible target range [105]. A systematic review and meta-analysis combining four prospective trials in this field, demonstrated that TDM for 5-FU reduces the risk of severe AEs, while improving the clinical response [106]. The benefit of TDM over screening is directly connecting drug level to clinical outcomes, with continued follow-up allowing for feedback and dose correction. However, TDM carries the risk of first cycle toxicity and therefore does not fully eliminate the need for pre-treatment screening for DPD deficiency. Other drawbacks of TDM are difficulties in standardizing the approach and the inherent costs of employing such an intensive program. Some constructive suggestions to address these concerns include centralized testing [107], and prospective cost-analysis to add to the very limited retrospective model-based literature [108]. Upon review of the available literature in this field, the International Association of Therapeutic Drug Monitoring and Clinical Toxicology released a guideline in favor the use of TDM for 5-FU [109]. However, TDM is not a clinical standard, and still requires prospective validation to confirm its efficacy and cost-effectiveness in modern fluoropyrimidine-based regimens. 

## 6. Clinical Dilemma

Given the complexity of this topic, it is not surprising that there are additional therapeutic dilemmas that are not accounted for in the literature. For example, within our center we operate a collaborative research program between the divisions of Clinical Pharmacology and Medical Oncology, in order to provide pre-treatment *DPYD* genotyping following the CPIC guidelines [28]. Recently we were requested to see a patient with planned fluoropyrimidine based therapy on the background of orthotopic liver transplant. This patient effectively possesses two genetic backgrounds. Given the liver serves as the primary site of 5-FU metabolism and we possessed no tissue to genotype, we were forced to go beyond our normal routine practice. We implemented TDM for this patient in real time with dose titration in accordance with published algorithms. The case report below details our process for implementing this without altering the treatment plan of the medical oncologists or delaying the patient’s treatment.

## 7. Case Presentation

A 40-year-old Caucasian male presented with painless jaundice and two-month history of bowel irregularity. The patient described loose stools, increasing in frequency over a two-month period, which floated and were difficult to flush. Past medical history is remarkable for a 14-year history of ulcerative colitis (UC), in remission, and Primary Sclerosing Cholangitis (PSC). At the time of presentation, the patient was two years post orthotopic liver transplant with curative intent for end stage liver disease secondary to rapid progression of his PSC. The patient tolerated the transplant well without acute rejection or infective complications. His medications included tacrolimus and prednisone. A routine abdominal ultrasound identified an irregular mass in the pancreas that led to additional imaging studies, including an abdominal computed tomography (CT). The abdominal CT with contrast identified a large, bulky, poorly delineated mass in the head of the pancreas. The mass was found to be invading segment 1 and 2 of the duodenum and obliterating the common bile duct. CT thorax and pelvis did not report metastatic disease. Magnetic resonance study confirmed locally advanced disease, deemed to be borderline resectable at initial presentation. An endoscopic ultrasound guided biopsy confirmed poorly differentiated adenocarcinoma of the pancreas. At this time, the case was reviewed by the multidisciplinary team and treatment options were presented to the patient. The patient, understanding the gravity of the diagnosis, wished to pursue maximal therapy and undergo neoadjuvant FOLFIRINOX followed by reassessment for potential curative resection. This triggered referral to our Personalized Medicine Clinic for *DPYD* genotype testing, the patient was genotyped using DNA from PBMCs and found to be wild-type for the following *DPYD* SNPs c.1905+1G>A, c.2846A>T, c.1679G>T, and c.1236G>A, tested in accordance with the CPIC guideline [28]. However, it was identified that given the patient’s history of orthotopic liver transplant of unknown *DPYD* status, there would be limited value in the genetic background of his PBMCs. Therefore, the treating medical oncologists proceeded with an initial dose reduction of 30% as a way of balancing the patient’s desire for maximal therapy and the care team’s desire to prevent early severe toxicity in this unknown setting. 

We planned to employ TDM utilizing liquid-liquid extraction and a high-pressure liquid chromatography tandem mass spectrometry assay developed in our laboratory for research purposes, to verify the patient’s systemic exposure was below the toxic threshold. Accordingly, for the first treatment of FOLFIRINOX, the patient received a 30% dose reduction of the 5-FU components. During the continuous infusion of 5-FU, a peripheral whole blood sample was collected from a venous puncture contralateral to the 5-FU infusion site. The sample was collected 2 h post initiation of the 5-FU continuous infusion pump. The sample was immediately placed on ice and the plasma was separated by centrifugation within 20 min at which time it was frozen to −80°C. We determined the patient’s plasma concentration of 5-FU to be 204.97 ng/mL, given a 46-h infusion this equates to an area under the curve of 9.43 mg·h/L, considered to be a subtherapeutic concentration. Combined with clinical observation of the patient, this result provided reassurance that the patient was not demonstrating signs of frank DPD deficiency. The treating oncologist utilized these results and titrated the dose accordingly while using published titration algorithms for reference [97,99]. The patient was keen to proceed to full dose intensity and the treating oncologists elected to administer the full dose of 5-FU with the reassurance of the TDM. To ensure this was an appropriate course of action and the transplant liver responded appropriately to the larger dose, we continued to monitor the patient. During the second cycle the patient was seen 24 h into the infusion instead of 2 h into continuous infusion as in the first cycle. Despite the known intra-patient variation changing the time of sampling was required to accommodate the logistics of this patient. The decision was deemed appropriate as the measurement would be at the predicted peak systemic 5-FU level and still serve to prevent supratherapeutic dosing. During the second infusion we found the patient’s plasma concentration of 5-FU to be 539.04 ng/mL, equating a predicted AUC of 24.8 mg·h/L. This falls directly within the known therapeutic range of 5-FU and provided confidence to the treating physician that the patient was now receiving optimal management with regards to the 5-FU component. The patient continued with FOLFIRINOX therapy, without developing any severe fluoropyrimidine-related AEs. Following this neoadjuvant course there was significant disease response and the patient proceeded to surgery with curative intent. 

## 8. Discussion

In this case, we have presented a therapeutic dilemma whereby a patient with a complex medical history and mixed genetic background, identified a limitation of pharmacogenetics. Upon review of the literature we believe there is a clinically important niche of orthotopic liver transplant patients where fluoropyrimidine therapy would benefit patient care. Immunosuppression post organ transplant induces an increased risk for development of neoplasms including skin, lymphoid and solid organ malignancies [110,111]. The increased rate of de novo colorectal, head and neck cancers is especially noteworthy as these disease sites are primary targets of fluoropyrimidine-based chemotherapy [112]. This patient’s medical history of ulcerative colitis and PSC pose additional risk factors for developing a de novo neoplasm. PSC is an aggressive disease often refractory to multiple therapies, ultimately the only curative treatment is orthotopic liver transplant [113]. PSC and ulcerative colitis are components of a constellation of diseases with an increased risk for the development of gastrointestinal malignancies [114]. PSC itself is directly related with an increased risk for solid tumour malignancies including cholangiocarcinoma, gall bladder carcinoma, colorectal cancer, and pancreatic adenocarcinoma [115]. With both an extensive history of ulcerative colitis and PSC this patient was a high-risk candidate to develop a post orthotopic liver transplant de novo neoplasm. Our patient developed pancreatic adenocarcinoma—known to have the highest mortality rate per case for malignant neoplasms—with median survival at diagnosis of 9 months [116]. Utilizing the most aggressive evidence-based approach in managing borderline resectable pancreatic adenocarcinoma, the treating oncologist used fluoropyrimidne-based chemotherapy in this complicated patient [117,118]. 

Unfortunately, due to the overall rarity of this condition, there is very little evidence for the effective use of fluoropyrimidines post orthotopic liver transplant. There is a limited body evidence for the use of fluoropyrimidines for adjuvant treatment in hepatocellular carcinoma treated with orthotopic liver transplant [119,120,121]. However, this data remains limited due to small sample size and the clinical preference for alternative treatment modalities in the treatment of hepatocellular carcinoma [122,123]. Therefore, there are no evidence-based recommendations for managing patients with this complex presentation. It is believed that practitioners should follow the same guidelines as with classical presentations [112]. This remains an intimidating dilemma owing to the known hepatotoxicity of fluoropyrimidines. There are case reports of both liver injury and graft rejection in liver transplant recipients receiving fluoropyrimidine-based chemotherapy [124,125]. These findings explain the caution with which the treating oncologists approached the care for this patient. We demonstrated that TDM in a post orthotopic liver transplant patient receiving 5-FU infusion was possible, and attainable within the normal timeline of therapy. The resultant information provided reassurance to the patient and practitioner without delaying therapy. TDM for 5-FU should be considered for fluoropyrimidine chemotherapy in orthotopic liver transplant recipients. The implementation of TDM for a unique case such as this underlies the benefit of combining pharmacogenetics and classic pharmacokinetic approaches to improve patient care through precision medicine.
